# The incidence, aetiology, and adverse clinical consequences of less severe diarrhoeal episodes among infants and children residing in low-income and middle-income countries: a 12-month case-control study as a follow-on to the Global Enteric Multicenter Study (GEMS)

**DOI:** 10.1016/S2214-109X(19)30076-2

**Published:** 2019-04-15

**Authors:** Karen L Kotloff, Dilruba Nasrin, William C Blackwelder, Yukun Wu, Tamer Farag, Sandra Panchalingham, Samba O Sow, Dipika Sur, Anita K M Zaidi, Abu S G Faruque, Debasish Saha, Pedro L Alonso, Boubou Tamboura, Doh Sanogo, Uma Onwuchekwa, Byomkesh Manna, Thandavarayan Ramamurthy, Suman Kanungo, Shahnawaz Ahmed, Shahida Qureshi, Farheen Quadri, Anowar Hossain, Sumon K Das, Martin Antonio, M Jahangir Hossain, Inacio Mandomando, Sozinho Acácio, Kousick Biswas, Sharon M Tennant, Jaco J Verweij, Halvor Sommerfelt, James P Nataro, Roy M Robins-Browne, Myron M Levine

**Affiliations:** a Center for Vaccine Development and Global Health, University of Maryland School of Medicine, Baltimore, MD, USA; b Department of Pediatrics, University of Maryland School of Medicine, Baltimore, MD, USA; c Department of Medicine, University of Maryland School of Medicine, Baltimore, MD, USA; d University of Maryland School of Medicine, Baltimore, MD, USA; e Centre pour le Développement des Vaccins, Bamako, Mali; f National Institute of Cholera and Enteric Diseases, Kolkata, India; g Department of Paediatrics and Child Health, the Aga Khan University, Karachi, Pakistan; h International Centre for Diarrhoeal Disease Research, Mohakhali, Dhaka, Bangladesh; i Medical Research Council Unit The Gambia at The London School of Hygiene & Tropical Medicine, Banjul, The Gambia; j Centro de Investigação em Saúde da Manhiça, Maputo, Mozambique; k Barcelona Institute for Global Health, Barcelona, Spain; l Barcelona Center for International Health Research, Barcelona, Spain; m Hospital Clínic-Universitat de Barcelona, Barcelona, Spain; n Department of Veterans Affairs, Cooperative Studies Program Coordinating Center, Perry Point, MD, USA; o Department of Parasitology, Leiden University Medical Center, Leiden, Netherlands; p Centre for Intervention Science in Maternal and Child Health, Department of Global Public Health and Primary Care, University of Bergen, Bergen, Norway; q Norwegian Institute of Public Health, Oslo, Norway; r Department of Microbiology and Immunology, The University of Melbourne, Parkville, VIC, Australia; s Murdoch Children's Research Institute, Royal Children's Hospital, Parkville, VIC, Australia; t Division of Microbiology & Immunity, Warwick Medical School, University of Warwick, Coventry, UK; u Instituto Nacional de Saúde, Ministério da Saúde, Maputo, Mozambique

## Abstract

**Background:**

Diarrheal diseases remain a leading cause of illness and death among children younger than 5 years in low-income and middle-income countries. The Global Enteric Multicenter Study (GEMS) has described the incidence, aetiology, and sequelae of medically attended moderate-to-severe diarrhoea (MSD) among children aged 0–59 months residing in censused populations in sub-Saharan Africa and south Asia, where most child deaths occur. To further characterise this disease burden and guide interventions, we extended this study to include children with episodes of less-severe diarrhoea (LSD) seeking care at health centres serving six GEMS sites.

**Methods:**

We report a 1-year, multisite, age-stratified, matched case-control study following on to the GEMS study. Six sites (Bamako, Mali; Manhiça, Mozambique; Basse, The Gambia; Mirzapur, Bangladesh; Kolkata, India; and Bin Qasim Town, Karachi, Pakistan) participated in this study. Children aged 0–59 months at each site who sought care at a sentinel hospital or health centre during a 12-month period were screened for diarrhoea. New (onset after ≥7 diarrhoea-free days) and acute (onset within the previous 7 days) episodes of diarrhoea in children who had sunken eyes, whose skin lost turgor, who received intravenous hydration, who had dysentery, or who were hospitalised were eligible for inclusion as MSD. The remaining new and acute diarrhoea episodes among children who sought care at the same health centres were considered LSD. We aimed to enrol the first eight or nine eligible children with MSD and LSD at each site during each fortnight in three age strata: infants (aged 0–11 months), toddlers (aged 12–23 months), and young children (aged 24–59 months). For each included case of MSD or LSD, we enrolled one to three community control children without diarrhoea during the previous 7 days. From patients and controls we collected clinical and epidemiological data, anthropometric measurements, and faecal samples to identify enteropathogens at enrolment, and we performed a follow-up home visit about 60 days later to ascertain vital status, clinical outcome, and interval growth. Primary outcomes were to characterise, for MSD and LSD, the pathogen-specific attributable risk and population-based incidence values, and to assess the frequency of adverse clinical consequences associated with these two diarrhoeal syndromes.

**Findings:**

From Oct 31, 2011, to Nov 14, 2012, we recruited 2368 children with MSD, 3174 with LSD, and one to three randomly selected community control children without diarrhoea matched to cases with MSD (n=3597) or LSD (n=4236). Weighted adjusted population attributable fractions showed that most attributable cases of MSD and LSD were due to rotavirus, *Cryptosporidium* spp, enterotoxigenic *Escherichia coli* encoding heat-stable toxin (with or without genes encoding heat-labile enterotoxin), and *Shigella* spp. The attributable incidence per 100 child-years for LSD versus MSD, by age stratum, for rotavirus was 22·3 versus 5·5 (0–11 months), 9·8 versus 2·9 (12–23 months), and 0·5 versus 0·2 (24–59 months); for *Cryptosporidium* spp was 3·6 versus 2·3 (0–11 months), 4·3 versus 0·6 (12–23 months), and 0·3 versus 0·1 (24–59 months); for enterotoxigenic *E coli* encoding heat-stable toxin was 4·2 versus 0·1 (0–11 months), 5·2 versus 0·0 (12–23 months), and 1·1 versus 0·2 (24–59 months); and for *Shigella* spp was 1·0 versus 1·3 (0–11 months), 3·1 versus 2·4 (12–23 months), and 0·8 versus 0·7 (24–59 months). Participants with both MSD and LSD had significantly more linear growth faltering than controls at follow-up.

**Interpretation:**

Inclusion of participants with LSD markedly expands the population of children who experience adverse clinical and nutritional outcomes from acute diarrhoeal diseases. Since MSD and LSD have similar aetiologies, interventions targeting rotavirus, *Shigella* spp, enterotoxigenic *E coli* producing heat-stable toxin, and *Cryptosporidium* spp might substantially reduce the diarrhoeal disease burden and its associated nutritional faltering.

**Funding:**

Bill & Melinda Gates Foundation.

## Introduction

The Global Enteric Multicenter Study (GEMS) was a prospective, age-stratified, matched case-control study of the burden, aetiology, and adverse clinical outcomes of diarrhoeal diseases among children aged 0–59 months seeking care at health-care facilities during a 36-month period at seven sites in sub-Saharan Africa and south Asia.^1^, ^2^ GEMS aimed to identify the most clinically severe, medically attended diarrhoeal episodes to guide and prioritise efforts to prevent the most life-threatening and disabling illnesses. It is also important to characterise the less-severe diarrhoea (LSD) episodes for which care is sought at health-care facilities, recognising that even though there might be fewer adverse health consequences from LSD, its overall burden could be greater than that of moderate-to-severe diarrhoea (MSD) because it is more common. Whether there are meaningful differences in the distribution of aetiologies of LSD compared with MSD must also be understood to optimise diarrhoeal disease prevention and treatment. Herein we describe a 1-year follow-on study designated GEMS-1A in which six GEMS sites did simultaneous case-control studies of both MSD and LSD to measure the pathogen-specific attributable risk and population-based incidence for LSD in addition to MSD—so the total burden of medically attended diarrhoeal disease in low-income and middle-income countries could be described—and to assess the frequency of adverse clinical consequences of these two syndromes.

## Methods

### Study design and participants

GEMS-1A is a 1-year, multisite, age-stratified, matched case-control study following on to the GEMS study.^1^ Six GEMS sites with moderate-to-high mortality of children younger than 5 years participated in GEMS-1A, three in Africa (Bamako, Mali; Manhiça, Mozambique; and Basse, The Gambia) and three in Asia (Mirzapur, Bangladesh; Kolkata, India; and Bin Qasim Town, Karachi, Pakistan).^1^ The estimated number of LSD cases at the GEMS site in Kenya was projected to be insufficient for participation in GEMS-1A. Participants at each site belonged to a censused population serially updated for births, deaths, and migrations using a demographic surveillance system (DSS). For participant enrolment, site investigators selected sentinel hospitals or health centres where children included in the DSS sought care for diarrhoeal illnesses.^1^

All children aged 0–59 months belonging to the DSS population at each site who sought care at a sentinel hospital or health centre during a 12-month period were screened for diarrhoea (three or more loose stools during the previous 24 h).^3^ Episodes eligible for inclusion as MSD were new (onset after ≥7 diarrhoea-free days) and acute (onset within the previous 7 days) episodes in children who satisfied at least one of these criteria: had sunken eyes (confirmed by parent or caretaker as more than normal); decreased skin turgor (abdominal skin pinch with slow or very slow [>2 s] recoil); intravenous hydration administered or prescribed; had dysentery (reported or visible blood in loose stools); or were hospitalised.^1^, ^2^ Eligibility was assessed by the child's clinician in conjunction with the study staff. The remaining new and acute diarrhoea episodes among children aged 0–59 months of age belonging to the DSS who sought care at the same health centres during the 12-month study period and did not meet the case definition of MSD were considered LSD. We aimed to enrol the first eight or nine eligible children with MSD and LSD at each site during each fortnight in three age strata: infants (aged 0–11 months), toddlers (aged 12–23 months), and young children (aged 24–59 months).^1^ For each included case of MSD or LSD, we enrolled one-to-three community control children without diarrhoea during the previous 7 days.^1^ Using a computer algorithm, at least four children were randomly selected from the site's DSS database among those who matched each individual enrolled patient by age, gender, and residence (same or nearby village or neighbourhood as the patient) according to predefined criteria.^1^ A field worker visited the homes of each selected child and sequentially enrolled, within 14 days of the diarrhoeal episode, the requisite number of children who met eligibility criteria.^1^

**Research in context**

**Evidence before this study**

Before this study, we did a systematic review of epidemiological studies seeking to determine the causes and adverse sequelae of paediatric diarrhoea in low-income countries. We searched PubMed for new studies and review articles published between Jan 1, 1980, and Aug 31, 2018, using the search string (“diarrhea*”[All Fields] OR “gastroenteritis”[All Fields]) AND (“pediatric”[All Fields] OR “child*”[All Fields]) AND (“*etiology”[All Fields] OR “growth faltering”[All Fields] OR “stunting”[All Fields]) AND (“developing countr*”[All Fields] OR “low-income”[All Fields]). We included older reports, and articles identified in reference lists when appropriate. We identified methodologic limitations that led to knowledge gaps about the epidemiology of diarrhoeal disease among children living in developing countries. We then designed and did the Global Enterics Multicenter Study (GEMS) to elucidate the incidence, aetiology, and adverse clinical consequences of medically attended moderate-to-severe diarrhoea (MSD) among children younger than 5 years living in developing countries. However, GEMS left unanswered questions about whether the findings of GEMS were generalisable to episodes of less-severe diarrhoea (LSD), which represent the majority of paediatric diarrhoea in patients presenting to health-care centres. Therefore, we updated our literature search and designed this study to simultaneously examine MSD and LSD.

**Added value of this study**

Using a rigorous study design and microbiological methods capable of detecting a broad array of pathogens across a diverse set of study sites with medium and high under-5 mortality, we showed that inclusion of LSD defines a far greater burden of disease without substantially altering the four most important aetiological pathogens involved—ie, rotavirus, *Cryptosporidium* spp, enterotoxigenic *Escherichia coli* producing heat-stable toxin, and *Shigella* spp. While children with LSD are less acutely ill than those with MSD, particularly with regard to dehydration, they have similar susceptibility to linear growth faltering following their diarrhoeal episode relative to their matched healthy controls.

**Implications of all the available evidence**

These findings expand the population of children experiencing adverse clinical and nutritional consequences of acute diarrhoeal illness in low-resource settings. Since MSD and LSD have similar aetiologies, mitigation of disease associated with a restricted number of aetiological agents can substantially reduce the diarrhoeal disease burden and its associated nutritional faltering.

The clinical protocol was approved by ethics committees at the University of Maryland (Baltimore, MD, USA) and those overseeing investigators at the field sites. Written informed consent was obtained from the parent or primary caretaker of each participant.

### Procedures

The primary outcomes of the GEMS-1A study were to characterise, for LSD in addition to MSD, the overall and pathogen-specific population-based attributable incidence and the pathogen-specific attributable fraction, and to assess the frequency of nutritional faltering and other adverse clinical consequences among children with these two diarrhoeal syndromes relative to the control population. The outcomes were assessed by site and age stratum, and across all sites for incidence and nutritional outcomes. Since dysentery was an exclusion criterion for LSD, we included a category non-dysentery MSD to compare syndromes of watery diarrhoea for analyses of attributable fraction and pathogen-specific incidence.

Other primary outcomes—eg, the mortality and frequency of persistent diarrhoea in children with LSD and MSD—will be published elsewhere.

GEMS-1A generally used the same clinical,^1^ epidemiological,^1^ microbiological,^4^ data management,^5^ and analytical^6^ methods described for GEMS, unless otherwise specified. At the time of these studies, no site had introduced rotavirus vaccine into its Expanded Programme on Immunization for infants.

To estimate population-based diarrhoeal disease, we did brief surveys on health-care utilisation and attitudes concurrent with the GEMS-1A case-control study using random samples of children.^7^ For children who had experienced diarrhoea in the previous 7 days, the primary caretaker was queried about clinical symptoms and health-care use for the episode. For each site and age stratum, we calculated the proportion of children who were taken to a sentinel hospital or health centre within 7 days of onset of diarrhoea and the pathogen-specific incidence per 100 child-years in the DSS population.^6^

At enrolment, parents or primary caretakers of all participants underwent standardised interviews to solicit demographic, epidemiological, and clinical information. GEMS staff measured each child's length or height.^1^ Medical management by clinicians at the sentinel hospital or health centre and clinical condition upon discharge were documented. A single follow-up home visit was done about 60 days after enrolment (range 50–90 days) to assess the child's vital status and repeat anthropometric measurements.

At enrolment, each participant provided fresh stool that was placed in cold storage and transport media according to the protocol.^1^ If antibiotics were to be administered to participants with diarrhoea before stool was produced, we obtained two rectal swabs for bacterial culture pending passage of the whole stool for the remaining assays.^1^

Putative enteropathogens (*Salmonella, Shigella, Campylobacter, Aeromonas*, and *Vibrio* spp, diarrhoeagenic *Escherichia coli* [enterotoxigenic, enteropathogenic, enteroaggregative, and Shiga toxin-producing], rotavirus, adenovirus serotypes 40 and 41, norovirus genotypes I and II, sapovirus, astrovirus, *Giardia intestinalis, Entamoeba histolytica*, and *Cryptosporidium* spp) were identified in cases and controls as previously described^4^ with some exceptions. *E coli* strains were first tested using a multiplex PCR as described for GEMS.^4^ An additional duplex PCR (appendix) was incorporated with primers to detect *E coli* encoding porcine heat-stable toxin and with alternative primers that detect *eae* but that generate a smaller *eae* amplicon than the first multiplex PCR. *E coli* that were *eae*^+^ and *bfp*^−^ were subsequently tested for *bfp* in a monoplex PCR. *E coli* that were *eae*^+^ and *bfp*^−^ were subsequently tested using a multiplex PCR with primers for *stx1, stx2, eae, efa-1* (enterohaemorrhagic *E coli*), and *sen* (enteropathogenic *E coli*). We also detected a *Helicobacter pylori* antigen by the Amplified IDEIA Hp StAR immunoassay (Oxoid, Thermofisher, Cambridge, UK), intestinal geohelminths (*Ascaris lumbricoides, Strongyloides stercoralis*, and human hookworms [*Necator americanus* and *Ancylostoma duodenale*]) using multiplex real-time PCR,^8^, ^9^ *Bacteroides fragilis* enterotoxin gene (*bftP*) by gel-based PCR on DNA extracted from stools,^10^ and *Clostridium difficile* using the C diff Quik Chek Complete dual antigen immunoassay (TechLab, Blacksburg VA, USA) by finding positivity to *C difficile* glutamate-dehydrogenase antigen and identifying the presence of toxins A or B.

### Statistical analysis

The analytic methods used in GEMS-1A followed those used in GEMS,^11^ with the additions described in this section. We used Wald χ^2^ tests to compare proportions of children with MSD or LSD and their matched controls with different demographic features. In separate analyses for MSD, non-dysentery MSD, and LSD, associations with potential pathogens were assessed with conditional logistic regression^12^ with a penalised likelihood approach.^13^ In brief, a weighted population attributable fraction^14^ for each pathogen significantly associated with MSD or LSD was derived for each site and age stratum from a multiple conditional logistic regression model that adjusted for the presence of other pathogens and interactions between pathogens. Pathogens were included in the multiple conditional logistic regression model if they were significant (p<0·1) in a bivariate analysis and remained after a process of backward elimination which used a prespecified p-value cutoff of 0·05. The pathogen-specific attributable fractions of non-dysentery MSD and LSD were compared by calculating a Z score for their difference, with the standard deviation of each attributable fraction estimated by jackknife.^15^

Once the attributable fraction for each pathogen was determined for each site and age stratum, we used the proportion of children with MSD or LSD taken to one of the site's sentinel hospitals or health centres—obtained from the data from the health-care utilisation and attitudes surveys—to calculate the pathogen-specific attributable incidence per 100 child-years in the DSS population.^6^ This method assumes that the distributions of aetiologies of MSD and LSD for children who sought care at the sentinel hospital or health centre were similar to the distributions for children who did not seek care. To assess this assumption, we used the data from the health-care utilisation and attitudes surveys to qualitatively compare the severity of illness as determined by caretakers' reports of the clinical features of children with MSD and LSD who did and did not seek care at a sentinel hospital or health centre.

For LSD, MSD, and non-dysentery MSD, overall pathogen-specific attributable incidence within each age group were calculated as the sum over sites of attributable cases (attributable fraction multiplied by total cases at sentinel hospitals or health centres and divided by the proportion of children with MSD or LSD taken to one of the site's sentinel hospitals or health centres), divided by the sum over sites of median DSS population. Standard errors of attributable incidence values were approximated using Taylor series to first derivative terms.

We derived length-for-age or height-for-age Z scores (HAZs) using WHO standards.^11^, ^16^ Weighted HAZ means at enrolment for patients and controls were compared using weighted paired *t* tests; when a patient had multiple controls, the average enrolment HAZ was used. The same weights used for the diarrhoeal aetiology analysis were used for the weighted paired *t* tests.^6^ These weights were also used in weighted linear regression analyses comparing baseline HAZ scores in patients with MSD and patients with LSD. We compared changes in HAZ from enrolment to follow-up in patients and controls using weighted linear regression models for all possible matched pairs, adjusting for enrolment HAZ and duration of follow-up and using jack-knife estimates of standard error.^15^

Results with two-sided p values less than 0·05 were considered significant. We did not apply any adjustment for multiple comparisons. Statistical analyses were performed in SAS version 9, SPSS version 24, and R version 3.3.2.

### Role of the funding source

The funder of the study played no role in study design, data collection, data analysis, data interpretation, or writing of the report. The corresponding author had full access to all the data in the study and had final responsibility for the decision to submit for publication.

## Results

**Figure 1:**
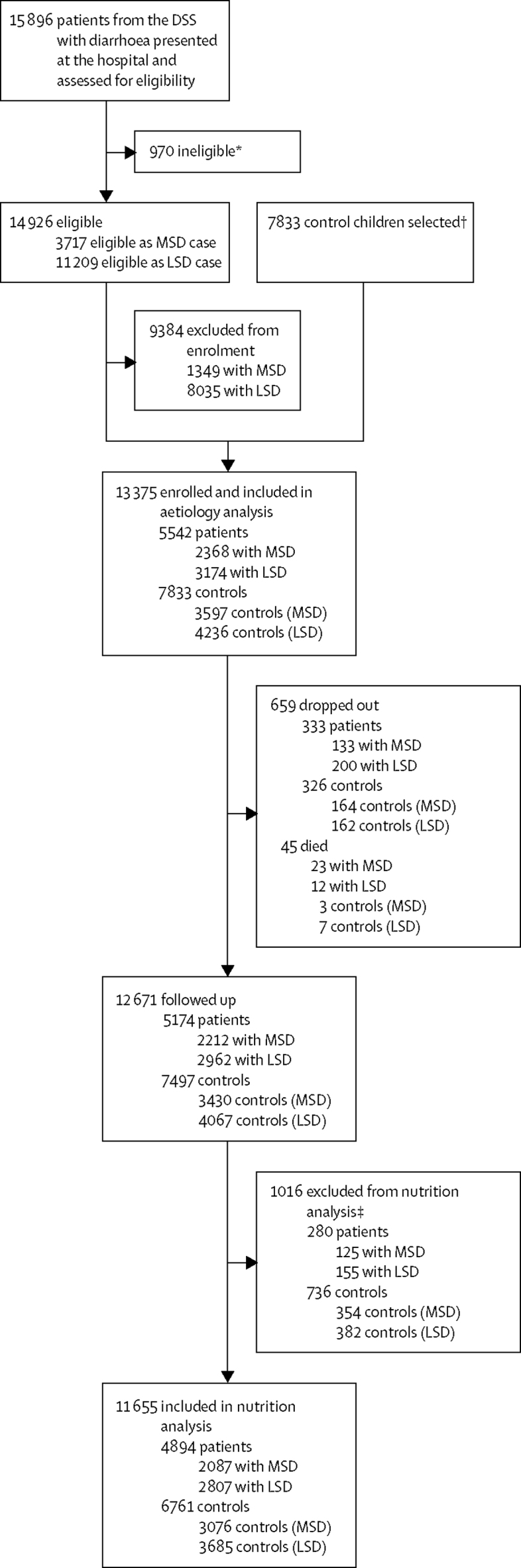
Study profile LSD=less-severe diarrhoea. MSD= moderate-to-severe diarrhoea. *Children were ineligible if their diarrheal episode had not started in the past 7 days after 7 diarrhea-free days, or if they were currently enrolled in the study and undergoing follow-up. †1–3 controls matched for age, gender, time of case presentation, and location of residence were selected randomly from the census database and given a stool collection kit; the first to produce a stool was enrolled; therefore, no controls were excluded. ‡Cases and controls were excluded from the nutritional analysis if they met criteria for an implausible value for height for age at enrollment or change in height over the follow-up period.

During a 12-month period between Oct 31, 2011, and Nov 14, 2012, children aged 0–59 months of age included in the DSS at six study sites (Bamako, Mali; Manhiça, Mozambique; Basse, The Gambia; Mirzapur, Bangladesh; Kolkata, India; and Bin Qasim Town, Karachi, Pakistan) collectively made 192 086 visits to the study sentinel hospitals or health centres, of which 15 896 (8·3%) were by children experiencing diarrhoea; 11 209 (75·1%) of the 14 926 children with acute, new onset diarrhea had LSD and 3717 (24·9%) had MSD. 2368 (63·7%) of 3717 children with MSD and 3174 (28·3%) of 11 209 with LSD were enrolled and analysable along with 3597 controls matched to patients with MSD and 4236 controls matched to patients with LSD (figure 1). 23 (1·0%) of 2368 children with MSD, 12 (0·4%) of 3174 children with LSD, three (<0·1%) of 3597 MSD controls, and seven (<0·1%) of 4236 LSD controls died after enrolment. Among living children, a 60-day follow-up household visit was completed for 2212 (94·3%) of 2345 children with MSD and 2962 (93·7%) of 3162 children with LSD, and for 3430 (95·4%) of 3594 MSD controls and 4067 (96·2%) of 4229 LSD controls. When we compared the demographic features of children with LSD (appendix) and MSD (appendix) to their matched controls, no trends were apparent. The proportion of MSD and LSD episodes (all sites combined) reported by caretakers during the health-care utilisation and attitudes surveys that met WHO criteria for dehydration was similar among children who did and did not seek care at a sentinel hospital or health centre (appendix).

**Figure 2:**
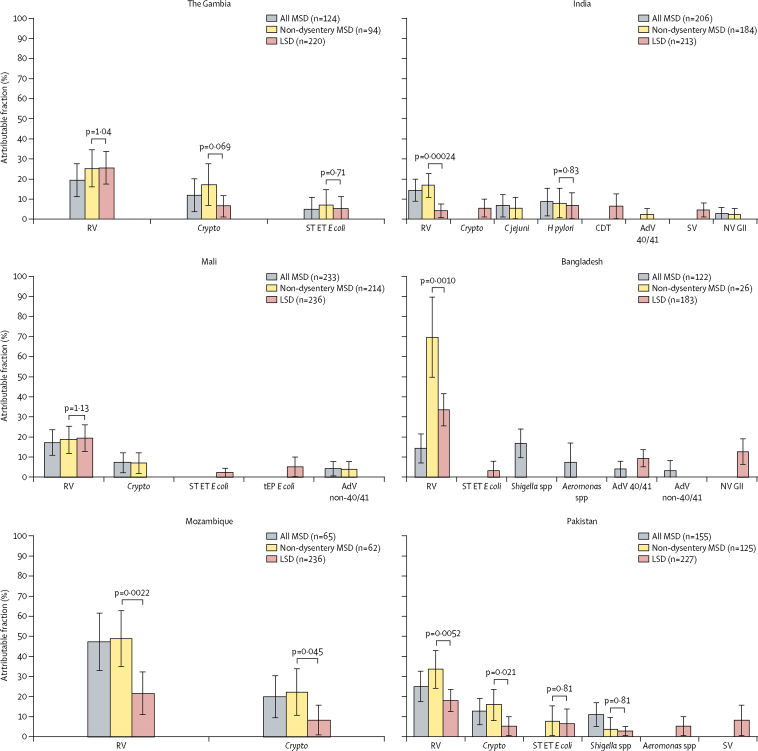
Adjusted attributable fraction of pathogens significantly associated with LSD, non-dysentery MSD, and MSD, by site in the 0–11 months' age group Adjusted attributable fractions are expressed as weighted percent of total diarrhoeal episodes. Bars are 95% CIs. Differences in pathogen frequency according to the severity of watery diarrhoea were evaluated by comparing non-dysentery MSD versus LSD using Z scores of the differences between non-dysentery MSD versus LSD. AdV=adenovirus. *C jejuni=Campylobacter jejuni*. CDT=*Clostridium difficile* toxin. *Crypto=Cryptosporidium* spp. *E coli=Escherichia coli*. ET=enterotoxigenic. *H pylori=Helicobacter pylori*. LSD=less-severe diarrhoea. MSD=moderate-to-severe diarrhoea. NV GII=norovirus GII. RV=rotavirus. ST=heat-stable-toxin producing. SV=sapovirus. tEP=typical enteropathogenic.

**Figure 3:**
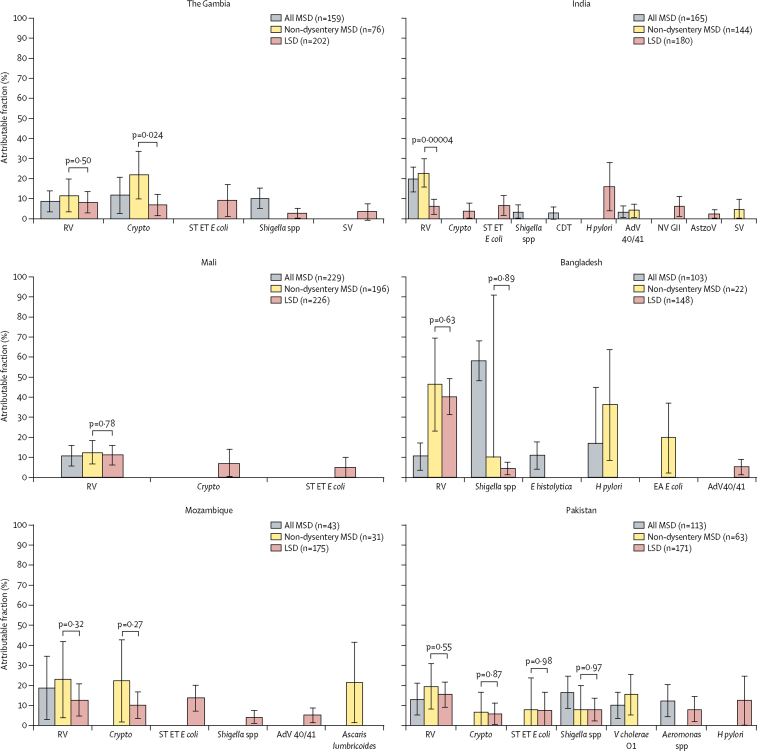
Adjusted attributable fraction of pathogens significantly associated with LSD, non-dysentery MSD, and MSD, by site in the 12–23 months' age group Adjusted attributable fractions are expressed as weighted percent of total diarrhoeal episodes. Bars are 95% CIs. Differences in pathogen frequency according to the severity of watery diarrhoea were evaluated by comparing non-dysentery MSD versus LSD using Z scores of the differences between non-dysentery MSD versus LSD. AdV=adenovirus. AstroV=astrovirus. CDT=*Clostridium difficile* toxin. *Crypto=Cryptosporidium* spp. EA=enteroaggregative. *E coli=Escherichia coli. E histolytica=Entamoeba histolytica*. ET=enterotoxigenic. *H pylori=Helicobacter pylori*. LSD=less-severe diarrhoea. MSD=moderate-to-severe diarrhoea. NV GII=norovirus GII. RV=rotavirus. ST=heat-stable-toxin producing. SV=sapovirus. *V cholerae=Vibrio cholerae*.

**Figure 4:**
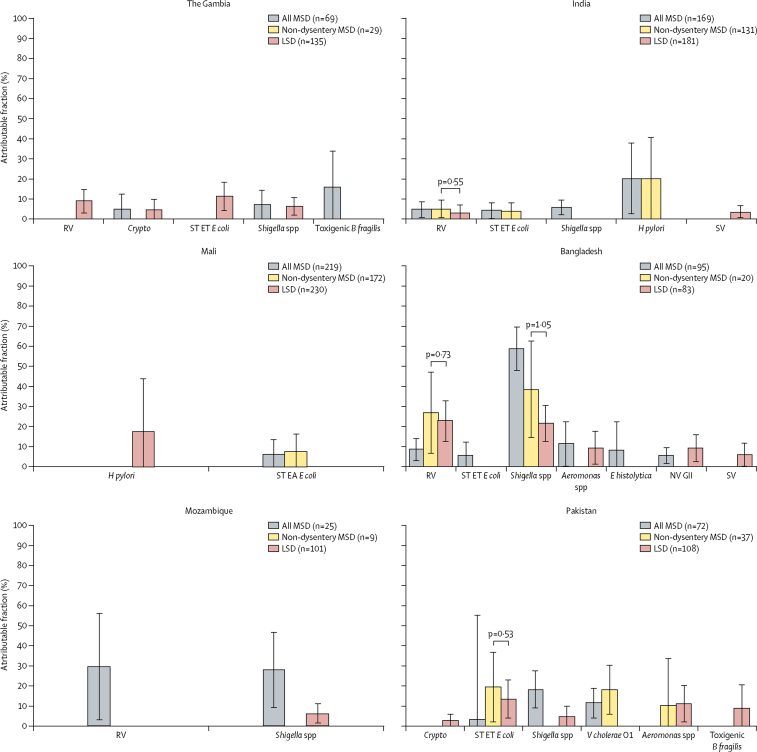
Adjusted attributable fraction of pathogens significantly associated with LSD, non-dysentery MSD, and MSD, by site in the 24–59 months' age group Adjusted attributable fractions are expressed as weighted percent of total diarrhoeal episodes. Bars are 95% CIs. Differences in pathogen frequency according to the severity of watery diarrhoea were evaluated by comparing non-dysentery MSD versus LSD using Z scores of the differences between non-dysentery MSD versus LSD. *B fragilis*=*Bacteroides fragilis. Crypto=Cryptosporidium* spp. *E coli=Escherichia coli. E histolytica=Entamoeba histolytica*. ET=enterotoxigenic. *H pylori=Helicobacter pylori*. LSD=less-severe diarrhoea. MSD=moderate-to-severe diarrhoea. NV GII=norovirus GII. RV=rotavirus. ST=heat-stable-toxin producing. SV=sapovirus. *V cholerae=Vibrio cholerae*.

Figure 2, figure 3, and figure 4 show the attributable fractions of the pathogens that were significantly associated with MSD, non-dysentery MSD, and LSD. During infancy (figure 2), rotavirus was the most common pathogen associated with non-dysentery MSD at every site, with overall MSD at all sites except Bangladesh (where *Shigella* was also an important cause of MSD), and with LSD at all sites except India. It predominated as a cause of MSD and LSD at all sites at age 12–23 months (figure 3), and at three sites each for MSD and LSD in the oldest stratum (figure 4). *Cryptosporidium* spp ranked second as a cause of MSD among infants at four sites (The Gambia, Mali, Mozambique, and Pakistan); among toddlers it ranked first or second in two sites (The Gambia [for both MSD and non-dysentery MSD] and Mozambique [for non-dysentery MSD]) and was significantly associated with LSD among infants at four sites (The Gambia, Mozambique, India, and Pakistan), toddlers at five sites (The Gambia, Mali, Mozambique, Pakistan, and India), and older children at two sites (The Gambia and Pakistan). The attributable fraction of *Shigella* spp increased with age and was significantly associated with MSD or LSD, or both, at two sites during infancy (Bangladesh and Pakistan) and five sites (all sites except Mali) in each of the older strata. Adenovirus 40 and 41 was associated with diarrhoea at three sites (India, Bangladesh, and Mozambique) and norovirus GII at two sites (India and Bangladesh). *H pylori* was significantly associated with diarrhoea at four sites; in India the association was seen in all age groups, while it was observed in a single age group in the other three sites (Pakistan, Bangladesh, and Mali).

Differences in pathogen frequency according to the severity of watery diarrhoea were evaluated by comparing non-dysentery MSD versus LSD. Significant differences were observed only in the two youngest age groups (figure 2; figure 3). Two pathogens were significantly more common in non-dysentery MSD compared to LSD: rotavirus at four sites (India, Bangladesh, Mozambique, and Pakistan) in the first year of life and at one site in the 12–23 month age group (India), and *Cryptosporidium* spp at two sites in the first year of life (Pakistan and Mozambique) and at one site in the second year of life (The Gambia). Some pathogens, such as *Vibrio cholerae* O1, *Aeromonas* spp, astrovirus, *Campylobacter jejuni*, toxigenic *C difficile*, and norovirus GII, were significantly associated with diarrhoea only in Asia. As a result, the diversity of pathogens appeared greater at the Asian sites compared with the African sites.

**Figure 5:**
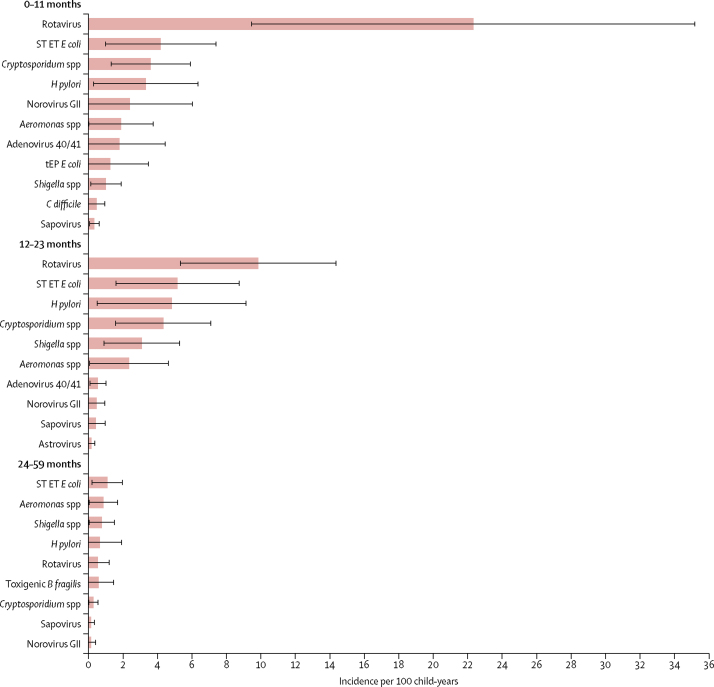
Attributable incidence of pathogen-specific LSD per 100 child-years of observation, by age stratum, all sites combined Bars show the incidence values and error bars show the 95% CIs. *B fragilis=Bacteroides fragilis. C difficile=Clostridium difficile. E coli=Escherichia coli*. ET=enterotoxigenic. *H pylori=Helicobacter pylori*. LSD=less-severe diarrhoea. ST=heat-stable-toxin producing. tEP=typical enteropathogenic.

**Figure 6:**
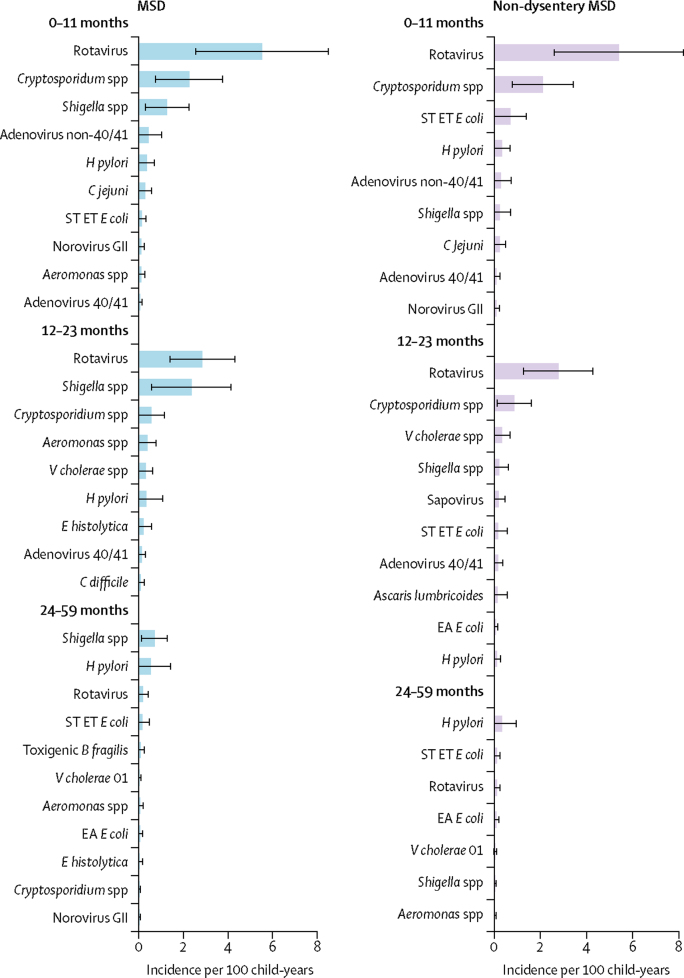
Attributable incidence of pathogen-specific moderate-to-severe diarrhoea (MSD) and non-dysentery MSD, per 100 child-years of observation, by age stratum, all sites combined Bars show the incidence values and error bars show the 95% CIs. *B fragilis=Bacteroides fragilis. C jejuni=Campylobacter jejuni. C difficile=Clostridium difficile*. EA=enteroaggregative. *E coli=Escherichia coli. E histolytica=Entamoeba histolytica*. ET=enterotoxigenic. *H pylori=Helicobacter pylori*. ST=heat-stable-toxin producing. *V cholerae=Vibrio cholerae*.

The overall incidence of LSD among infants from the DSS population (105·0 episodes per 100 child-years, 95% CI 53·4–156·6) was 3·8 times higher than that of MSD (27·7 episodes per 100 child-years, 13·2–42·3), and 4·8 times higher than that of non-dysentery MSD (21·7 episodes per 100 child-years, 10·0–33·5; table 1). When the pathogen-specific attributable incidence was examined across the six sites, the incidence of rotavirus was highest in relation to the other pathogens for LSD (22·3 episodes per 100 child-years, 9·4–35·2) and non-dysenteric MSD (5·4 episodes per 100 child-years, 2·6–8·2) during infancy, and continued to prevail among toddlers at a lower level (figure 5; figure 6). *Cryptosporidium* spp contributed the second highest incidence of non-dysenteric MSD among infants (2·1 episodes per 100 child-years, 0·8–3·4) and toddlers (0·9 episodes per 100 child-years, 0·1–1·6), while ranking third among infants and fourth among toddlers as a cause of LSD. *Shigella* spp had the third highest incidence for MSD among infants (1·3 episodes per 100 child-years, 0·3–2·3), the second among toddlers (2·4 episodes per 100 child-years, 0·6–4·1), and the first among older children (0·7 episodes per 100 child-years, 0·1–1·3); it ranked lower as a cause of non-dysenteric MSD and LSD. The incidence of MSD and non-dysenteric MSD caused by enterotoxigenic *E coli* producing heat-stable toxin was less than 0·2 per 100 child-years in all groups except infants with non-dysenteric MSD (0·7 episodes per 100 child-years, 0·0–1·4); by contrast, the incidence of LSD caused by enterotoxigenic *E coli* producing heat-stable toxin was 4·2 per 100 child-years (1·0–7·4) among infants, 5·2 per 100 child-years (1·6–8·7) among toddlers, and 1·1 per 100 child-years (0·2–2·0) among young children, ranking second to rotavirus in the infant and toddler groups and ranking first among the young-children group. One notable finding is the appearance of *H pylori* in the top five ranking agents in nearly all age groups.

**Table 1:** Incidence of moderate–to–severe diarrhoea (MSD) and less–severe diarrhoea (LSD) per 100–child–years of observation by site and age stratum

	**The Gambia**	**Mali**	**Mozambique**	**India**	**Bangladesh**	**Pakistan**	**Total**
**0–11 months**							
LSD	67·9 (17·1–118·6)	98·3 (0·0–242·3)	65·6 (0·0–175·9)	82·6 (45·1–120·2)	118·4 (0·0–290·6)	161·4 (82·1–240·7)	105·0 (53·4–156·6)
Total MSD	15·2 (3·2–27·1)	35·7 (0·0–82·2)	5·2 (0·0–13·6)	51·7 (24·8–78·5)	9·7 (1·2–18·3)	43·1 (10·2–76·0)	27·7 (13·2–42·3)
Non–dysenteric MSD	12·2 (2·0–22·4)	29·3 (0·0–67·4)	4·3 (0·0–11·3)	50·7 (22·6–78·7)	1·7 (0·2–3·3)	32·2 (7·6–56·7)	21·7 (10·0–33·5)
LSD + MSD	83·0 (30·9–135·2)	134·0 (0·0–285·3)	70·8 (0·0–181·5)	134·3 (88·2–180·5)	128·1 (0·0–300·5)	204·4 (118·6–290·3)	132·7 (79·1–186·3)
**12–23 months**							
LSD	48·8 (14·9–82·7)	57·6 (0·0–143·2)	53·2 (0·0–109·5)	89·6 (27·4–151·8)	23·3 (2·3–44·4)	189·4 (71·7–307·1)	72·2 (42·8–101·5)
Total MSD	20·5 (5·3–35·7)	33·6 (0·0–72·3)	6·7 (0·0–16·4)	51·5 (10·4–92·6)	11·5 (0·0–27·1)	21·0 (5·7–36·2)	23·2 (11·9–34·4)
Non–dysenteric MSD	11·5 (2·7–20·4)	25·2 (0·0–54·6)	6·1 (0·0–23·0)	52·8 (5·8–99·7)	1·8 (0·0–4·1)	14·7 (3·6–25·7)	16·4 (7·6–25·1)
LSD + MSD	69·3 (32·1–106·4)	91·2 (0·0–185·2)	59·9 (2·8–117·1)	141·1 (66·5–215·7)	34·8 (8·6–61·0)	210·4 (91·7–329·0)	95·3 (63·9–126·8)
**24–59 months**							
LSD	8·2 (1·6–14·7)	17·6 (0·0–38·6)	9·8 (0·0–24·7)	21·0 (9·0–33·0)	7·8 (0·0–23·1)	32·9 (10·2–55·5)	16·3 (9·0–23·6)
Total MSD	3·1 (0·2–5·9)	5·7 (0·3–11·1)	1·0 (0·0–2·7)	30·5 (0·0–73·2)	3·8 (0·0–8·7)	2·5 (0·7–4·4)	5·9 (1·8–9·9)
Non–dysenteric MSD	1·7 (0·0–3·5)	5·5 (0·7–10·3)	0·5 (0·0–1·4)	20·2 (0·0–48·5)	0·6 (0·0–1·3)	1·4 (0·4–2·5)	3·8 (1·1–6·5)
LSD + MSD	11·2 (4·1–18·4)	23·3 (1·6–45·0)	10·8 (0·0–25·8)	51·5 (7·1–95.8)	11·6 (0·0–27·7)	35·4 (12·7–58·1)	22·2 (13·8–30·6)

Data are incidence per 100 child-years (95% CI).

At enrolment, the weighted mean HAZ of both patients and controls was well below the WHO reference value; however, patients with both LSD (table 2) and MSD (table 3) had similar HAZs to their matched controls (all sites combined within age groups), with the exception of the infant stratum in the MSD analysis (table 3). At the follow-up visit, patients in the two highest age strata had significantly more linear growth faltering than controls after LSD and after MSD (table 2; table 3).

**Table 2:** Comparison of enrolment HAZ and ΔHAZ between patients with less severe diarrhoea and their matched controls, by site

		**0–11 months**		**12–23 months**		**24–59 months**	
		Weighted mean (95% CI)	p value	Weighted mean (95% CI)	p value	Weighted mean (95% CI)	p value
**Basse, The Gambia**							
Number of participants		199 patients; 228 controls	..	183 patients; 236 controls	..	120 patients; 212 controls	..
Enrolment HAZ							
	Patients	−0·56 (−0·74 to −0·38)	0·23	−1·21 (−1·39 to −1·03)	0·73	−1·43 (−1·63 to −1·23)	0·69
	Controls	−0·44 (−0·62 to −0·27)	..	−1·19 (−1·35 to −1·04)	..	−1·45 (−1·60 to −1·29)	..
ΔHAZ							
	Patients	−0·27 (−0·35 to −0·20)	0·67	−0·23 (−0·28 to −0·17)	0·14	−0·10 (−0·15 to −0·05)	0·10
	Controls	−0·23 (−0·31 to −0·14)	..	−0·14 (−0·19 to −0·09)	..	0·01 (−0·03 to 0·05)	..
**Bamako, Mali**							
Number of participants		204 patients; 204 controls	..	201 patients; 202 controls	..	208 patients; 208 controls	..
Enrolment HAZ							
	Patients	−0·57 (−0·72 to −0·41)	0·81	−0·88 (−1·04 to −0·72)	0·10	−1·07 (−1·22 to −0·93)	0·17
	Controls	−0·63 (−0·81 to −0·46)	..	−1·06 (−1·21 to −0·91)	..	−0·92 (−1·07 to −0·77)	..
ΔHAZ							
	Patients	−0·37 (−0·44 to −0·31)	0·31	−0·08 (−0·11 to −0·04)	0·39	0·06 (0·03 to 0·09)	0·18
	Controls	−0·32 (−0·39 to −0·25)	..	−0·04 (−0·09 to 0·01)	..	0·07 (0·05 to 0·10)	..
**Manhiça, Mozambique**							
Number of participants		136 patients; 136 controls	..	148 patients; 148 controls	..	81 patients; 81 controls	..
Enrolment HAZ							
	Patients	−0·83 (−1·04 to −0·63)	0·61	−1·30 (−1·51 to −1·10)	0·033	−1·52 (−1·80 to −1·24)	0·56
	Controls	−0·91 (−1·10 to −0·73)	..	−1·57 (−1·75 to −1·39)	..	−1·63 (−1·86 to −1·40)	..
ΔHAZ							
	Patients	−0·03 (−0·15 to 0·08)	0·64	−0·04 (−0·11 to 0·04)	0·65	−0·03 (−0·09 to 0·02)	0·020
	Controls	−0·07 (−0·18 to 0·04)	..	−0·03 (−0·11 to 0·05)	..	0·05 (0·00 to 0·11)	..
**Kolkata, India**							
Number of participants		194 patients; 194 controls	..	171 patients; 183 controls	..	175 patients; 181 controls	..
Enrolment HAZ							
	Patients	−1·12 (−1·28 to −0·97)	0·95	−1·47 (−1·64 to −1·30)	0·28	−1·74 (−1·91 to 1·56)	0·39
	Controls	−1·13 (−1·28 to −0·98)	..	−1·29 (−1·44 to −1·15)	..	−1·64 (−1·80 to −1·47)	..
ΔHAZ							
	Patients	−0·10 (−0·15 to −0·06)	0·12	−0·07 (−0·09 to −0·04)	0·42	−0·03 (−0·04 to −0·02)	0·72
	Controls	−0·05 (−0·09 to −0·01)	..	−0·02 (−0·06 to 0·01)	..	−0·03 (−0·04 to −0·01)	..
**Mirzapur, Bangladesh**							
Number of participants		176 patients; 344 controls	..	146 patients; 287 controls	..	82 patients; 243 controls	..
Enrolment HAZ							
	Patients	−1·06 (−1·26 to −0·85)	0·60	−1·05 (−1·23 to −0·88)	0·13	−0·98 (−1·20 to −0·76)	0·0058
	Controls	−1·05 (−1·19 to −0·92)	..	−1·32 (−1·44 to −1·19)	..	−1·43 (−1·56 to −1·30)	..
ΔHAZ							
	Patients	−0·21 (−0·29 to −0·14)	0·67	−0·16 (−0·22 to −0·10)	0·24	−0·09 (−0·13 to −0·05)	0·28
	Controls	−0·23 (−0·27 to −0·18)	..	−0·11 (−0·15 to −0·08)	..	−0·05 (−0·07 to −0·02)	..
**Karachi (Bin Qasim Town), Pakistan**							
Number of participants		161 patients; 162 controls	..	133 patients; 219 controls	..	89 patients; 217 controls	..
Enrolment HAZ							
	Patients	−1·31 (−1·50 to −1·12)	0·71	−2·04 (−2·27 to −1·81)	0·18	−2·34 (−2·67 to −2·02)	0·73
	Controls	−1·26 (−1·49 to −1·03)	..	−1·97 (−2·13 to −1·81)	..	−2·28 (−2·45 to −2·11)	..
ΔHAZ							
	Patients	−0·09 (−0·19 to 0·01)	0·18	−0·28 (−0·36 to −0·20)	0·0012	−0·04 (−0·11 to 0·02)	0·0043
	Controls	−0·16 (−0·26 to −0·06)	..	−0·16 (−0·21 to −0·10)	..	0·06 (0·02 to 0·09)	..
**All sites combined**							
Number of participants		1070 patients; 1268 controls	..	982 patients; 1275 controls	..	755 patients; 1142 controls	..
Enrolment HAZ							
	Patients	−0·98 (−1·07 to −0·88)	0·70	−1·47 (−1·58 to −1·37)	0·96	−1·79 (−1·94 to −1·64)	0·87
	Controls	−0·95 (−1·04 to −0·85)	..	−1·55 (−1·63 to −1·47)	..	−1·88 (−1·98 to −1·77)	..
ΔHAZ							
	Patients	−0·16 (−0·21 to −0·12)	0·23	−0·18 (−0·21 to −0·15)	0·0040	−0·04 (−0·07 to −0·01)	<0·0001
	Controls	−0·18 (−0·22 to −0·14)	..	−0·11 (−0·14 to −0·09)	..	0·03 (0·01 to 0·55)	..

Enrolment HAZ in patients versus controls was compared by weighted paired *t* test; ΔHAZ in patients versus controls was compared by weighted linear regression, adjusting for enrolment HAZ and duration to follow–up. HAZ=length–for–age or height–for–age Z score. ΔHAZ=change in HAZ (ie, HAZ at follow–up visit [50–90 days after enrolment] minus HAZ at enrolment).

**Table 3:** Comparison of enrolment HAZ and ΔHAZ between patients with moderate–to–severe diarrhoea and their matched controls, by site

		**0–11 months**		**12–23 months**		**24–59 months**	
		Weighted mean (95% CI)	p value	Weighted mean (95% CI)	p value	Weighted mean (95% CI)	p value
**Basse, The Gambia**							
Number of participants		103 patients; 155 controls	..	131 patients; 199 controls	..	62 patients; 135 controls	..
Enrolment HAZ							
	Patients	−0·90 (−1·12 to −0·68)	0·30	−1·53 (−1·73 to −1·33)	0·26	−1·82 (−2·08 to −1·55)	0·30
	Controls	−0·70 (−0·90 to −0·50)	..	−1·35 (−1·54 to −1·15)	..	−1·53 (−1·69 to −1·36)	..
ΔHAZ							
	Patients	−0·37 (−0·47 to −0·26)	0·93	−0·30 (−0·37 to −0·24)	0·091	−0·03 (−0·10 to 0·05)	0·57
	Controls	−0·20 (−0·29 to −0·12)	..	−0·09 (−0·15 to −0·03)	..	0·08 (0·04 to 0·13)	..
**Bamako, Mali**							
Number of participants		206 patients; 206 controls	..	206 patients; 206 controls	..	191 patients; 191 controls	..
Enrolment HAZ							
	Patients	−0·50 (−0·64 to −0·36)	0·31	−1·15 (−1·29 to −1·00)	0·67	−1·18 (−1·35 to −1·00)	0·41
	Controls	−0·57 (−0·76 to −0·38)	..	−1·04 (−1·18 to −0·90)	..	−1·06 (−1·22 to −0·89)	..
ΔHAZ							
	Patients	−0·40 (−0·45 to −0·34)	0·17	−0·09 (−0·13 to −0·05)	0·14	0·06 (0·03 to 0·09)	0·18
	Controls	−0·34 (−0·41 to −0·27)	..	−0·04 (−0·08 to 0·00)	..	0·11 (0·08 to 0·14)	..
**Manhiça, Mozambique**							
Number of participants		55 patients; 145 controls	..	37 patients;101 controls	..	22 patients; 60 controls	..
Enrolment HAZ							
	Patients	−1·39 (−1·77 to −1·01)	0·050	−1·66 (−2·18 to −1·14)	0·62	−1·07 (−1·59 to −0·55)	0·033
	Controls	−0·74 (−0·91 to −0·57)	..	−1·52 (−1·74 to −1·30)	..	−1·45 (−2·02 to −0·88)	..
ΔHAZ							
	Patients	−0·01 (−0·16 to −0·15)	0·62	−0·27 (−0·39 to −0·14)	0·038	0·10 (−0·04 to 0·23)	0·72
	Controls	−0·08 (−0·17 to −0·02)	..	−0·01 (−0·09 to 0·07)	..	0·08 (0·01 to 0·15)	..
**Kolkata, India**							
Number of participants		189 patients; 190 controls	..	147 patients; 160 controls	..	163 patients; 186 controls	..
Enrolment HAZ							
	Patients	−1·12 (1·26 to −0·98)	0·70	−1·30 (−1·48 to −1·12)	0·66	−1·90 (−2·09 to −1·71)	0·29
	Controls	−1·14 (−1·30 to −0·98)	..	−1·44 (−1·65 to −1·24)	..	−1·61 (−1·76 to −1·45)	..
ΔHAZ							
	Patients	−0·07 (−0·11 to −0·02)	0·41	−0·06 (−0·09 to −0·04)	0·47	−0·01 (−0·02 to 0·01)	0·87
	Controls	−0·02 (−0·06 to 0·02)	..	−0·04 (−0·07 to −0·01)	..	−0·01 (−0·02 to 0·00)	..
**Mirzapur, Bangladesh**							
Number of participants		121 patients; 241 controls	..	102 patients; 201 controls	..	93 patients; 270 controls	..
Enrolment HAZ							
	Patients	−1·05 (−1·24 to −0·85)	0·071	−1·30 (−1·53 to −1·06)	0·95	−1·29 (−1·51 to −1·07)	0·52
	Controls	−1·00 (−1·15 to −0·86)	..	−1·31 (−1·47 to −1·15)	..	−1·42 (−1·55 to −1·29)	..
ΔHAZ							
	Patients	−0·20 (−0·30 to −0·10)	0·36	−0·11 (−0·16 to −0·06)	0·51	−0·09 (−0·13 to −0·05)	0·016
	Controls	−0·25 (−0·31 to −0·20)	..	−0·07 (−0·12 to −0·03)	..	−0·05 (−0·08 to −0·03)	..
**Karachi (Bin Qasim Town), Pakistan**							
Number of participants		108 patients; 108 controls	..	89 patients; 163 controls	..	62 patients; 159 controls	..
Enrolment HAZ							
	Patients	−1·68 (−1·92 to −1·43)	0·065	−2·08 (−2·33 to −1·83)	0·84	−2·55 (−2·89 to −2·22)	0·42
	Controls	−1·24 (−1·48 to −1·00)	..	−2·03 (−2·23 to −1·83)	..	−2·32 (−2·52 to −2·13)	..
ΔHAZ							
	Patients	−0·17 (−0·29 to −0·05)	0·88	−0·20 (−0·31 to −0·10)	0·26	−0·09 (−0·17 to −0·02)	0·071
	Controls	−0·22 (−0·35 to −0·10)	..	−0·12 (−0·20 to −0·04)	..	0·01 (−0·04 to 0·07)	..
**All sites combined**							
Number of participants		782 patients; 1045 controls	..	712 patients; 1030 controls	..	593 patients; 1001 controls	..
Enrolment HAZ							
	Patients	−1·07 (−1·16 to −0·98)	0·030	−1·50 (−1·59 to −1·40)	0·31	−1·69 (−1·81 to −1·57)	0·35
	Controls	−0·89 (−0·97 to −0·82)	..	−1·48 (−1·57 to −1·40)	..	−1·65 (−1·73 to −1·56)	..
ΔHAZ							
	Patients	−0·23 (−0·27 to −0·19)	0·41	−0·17 (−0·21 to −0·14)	0·0002	−0·02 (−0·04 to 0·00)	0·0061
	Controls	−0·19 (−0·23 to −0·16)	..	−0·07 (−0·10 to −0·05)	..	0·02 (0·00 to 0·04)	..

Enrolment HAZ in patients versus controls was compared by weighted paired *t* test; ΔHAZ in patients versus controls was compared by weighted linear regression, adjusting for enrolment HAZ and duration to follow–up. HAZ=length–for–age or height–for–age Z score. ΔHAZ=change in HAZ (ie, HAZ at follow–up visit [50–90 days after enrolment] minus HAZ at enrolment).

Children with MSD had significantly lower enrolment mean HAZs than those with LSD in seven of the 18 site-specific age strata (ie, three age strata in six sites for a total of 18 strata; table 4). However, when all sites were combined in age stratum-specific analyses, significant differences were no longer apparent (table 4). Children with MSD had significantly more growth faltering over the approximately 60-day follow-up period than children with LSD in four of the 18 strata. When all sites were combined, a significant difference was seen in the infant stratum (table 4).

**Table 4:** Comparison of enrolment HAZ and ΔHAZ between patients with LSD and patients with MSD, by site.

		**0–11 months**		**12–23 months**		**24–59 months**	
		Weighted mean (95% CI)	p value	Weighted mean (95% CI)	p value	Weighted mean (95% CI)	p value
**Basse, The Gambia**							
Number of participants		199 LSD patients; 103 MSD patients	..	183 LSD patients; 131 MSD patients	..	120 LSD patients; 62 MSD patients	..
Enrolment HAZ							
	LSD patients	−0·56 (−0·74 to −0·38)	0·020	−1·21 (−1·39 to −1·03)	0·020	−1·43 (−1·63 to −1·23)	0·023
	MSD patients	−0·90 (−1·12 to −0·68)	..	−1·53 (−1·73 to −1·33)	..	−1·82 (−2·08 to −1·55)	..
ΔHAZ							
	LSD patients	−0·27 (−0·35 to −0·20)	0·038	−0·23 (−0·28 to −0·17)	0·013	−0·10 (−0·15 to −0·05)	0·22
	MSD patients	−0·37 (−0·47 to −0·26)	..	−0·30 (−0·37 to −0·24)	..	−0·03 (−0·10 to 0·05)	..
**Bamako, Mali**							
Number of participants		204 LSD patients; 206 MSD patients	..	201 LSD patients; 206 MSD patients	..	208 LSD patients; 191 MSD patients	..
Enrolment HAZ							
	LSD patients	−0·57 (−0·72 to −0·41)	0·52	−0·88 (−1·04 to −0·72)	0·015	−1·07 (−1·22 to −0·93)	0·38
	MSD patients	−0·50 (−0·64 to −0·36)	..	−1·15 (−1·29 to −1·00)	..	−1·18 (−1·35 to −1·00)	..
ΔHAZ							
	LSD patients	−0·37 (−0·44 to −0·31)	0·85	−0·08 (−0·11 to −0·04)	0·16	0·06 (0·03 to 0·09)	0·80
	MSD patients	−0·40 (−0·45 to −0·34)	..	−0·09 (−0·13 to −0·05)	..	0·06 (0·03 to 0·09)	..
**Manhiça, Mozambique**							
Number of participants		136 LSD patients; 55 MSD patients	..	148 LSD patients; 37 MSD patients	..	81 LSD patients; 22 MSD patients	..
Enrolment HAZ							
	LSD patients	−0·83 (−1·04 to −0·63)	0·010	−1·30 (−1·51 to −1·10)	0·19	−1·52 (−1·80 to −1·24)	0·12
	MSD patients	−1·39 (−1·77 to −1·01)	..	−1·66 (−2·18 to −1·14)	..	−1·07 (−1·59 to −0·55)	..
ΔHAZ							
	LSD patients	−0·03 (−0·15 to −0·08)	0·70	−0·04 (−0·11 to −0·04)	0·0020	−0·03 (−0·09 to 0·02)	0·033
	MSD patients	−0·01 (−0·16 to −0·15)	..	−0·27 (−0·39 to −0·14)	..	0·10 (−0·04 to 0·23)	..
**Kolkata, India**							
Number of participants		194 LSD patients; 189 MSD patients	..	171 LSD patients; 147 MSD patients	..	175 LSD patients; 163 MSD patients	..
Enrolment HAZ							
	LSD patients	−1·12 (−1·28 to −0·97)	0·94	−1·47 (−1·64 to −1·30)	0·18	−1·74 (−1·91 to 1·56)	0·21
	MSD patients	−1·12 (1·26 to −0·98)	..	−1·30 (−1·48 to −1·12)	..	−1·90 (−2·09 to −1·71)	..
ΔHAZ							
	LSD patients	−0·10 (−0·15 to −0·06)	0·27	−0·07 (−0·09 to −0·04)	0·80	−0·03 (−0·04 to −0·02)	0·20
	MSD patients	−0·07 (−0·11 to −0·02)	..	−0·06 (−0·09 to −0·04)	..	−0·01 (−0·02 to 0·01)	..
**Mirzapur, Bangladesh**							
Number of participants		176 LSD patients; 121 MSD patients	..	146 LSD patients; 102 MSD patients	..	82 LSD patients; 93 MSD patients	..
Enrolment HAZ							
	LSD patients	−1·06 (−1·26 to −0·85)	0·94	−1·05 (−1·23 to −0·88)	0·10	−0·98 (−1·20 to −0·76)	0·045
	MSD patients	−1·05 (−1·24 to −0·85)	..	−1·30 (−1·53 to −1·06)	..	−1·29 (−1·51 to −1·07)	..
ΔHAZ							
	LSD patients	−0·21 (−0·29 to −0·14)	0·95	−0·16 (−0·22 to −0·10)	0·48	−0·09 (−0·13 to −0·05)	0·69
	MSD patients	−0·20 (−0·30 to −0·10)	..	−0·11 (−0·16 to −0·06)	..	−0·09 (−0·13 to −0·05)	..
**Karachi (Bin Qasim Town), Pakistan**							
Number of participants		161 LSD patients; 108 MSD patients	..	133 LSD patients; 89 MSD patients	..	89 LSD patients; 62 MSD patients	..
Enrolment HAZ							
	LSD patients	−1·31 (−1·50 to −1·12)	0·021	−2·04 (−2·27 to −1·81)	0·82	−2·34 (−2·67 to −2·02)	0·38
	MSD patients	−1·68 (−1·92 to −1·43)	..	−2·08 (−2·33 to −1·83)	..	−2·55 (−2·89 to −2·22)	..
ΔHAZ							
	LSD patients	−0·09 (−0·19 to 0·01)	0·25	−0·28 (−0·36 to −0·20)	0·16	−0·04 (−0·11 to 0·02)	0·090
	MSD patients	−0·17 (−0·29 to −0·05)	..	−0·20 (−0·31 to −0·10)	..	−0·09 (−0·17 to −0·02)	..
**All sites combined**							
Number of participants		1070 LSD patients; 782 MSD patients	..	982 LSD patients; 712 MSD patients	..	755 LSD patients; 593 MSD patients	..
Enrolment HAZ							
	LSD patients	−0·98 (−1·07 to −0·88)	0·17	−1·47 (−1·58 to −1·37)	0·78	−1·79 (−1·94 to −1·64)	0·30
	MSD patients	−1·07 (−1·16 to −0·98)	..	−1·50 (−1·59 to −1·40)	..	−1·69 (−1·81 to −1·57)	..
ΔHAZ							
	LSD patients	−0·16 (−0·21 to −0·12)	0·018	−0·18 (−0·21 to −0·15)	0·77	−0·04 (−0·07 to −0·009)	0·19
	MSD patients	−0·23 (−0·27 to −0·19)	..	−0·17 (−0·21 to −0·14)	..	−0·02 (−0·04 to 0·003)	..

Enrolment HAZ in patients with LSD versus patients with MSD was compared by weighted linear regression. ΔHAZ was compared by weighted linear regression, adjusting for enrolment HAZ and duration to follow–up. HAZ=length–for–age or height–for–age Z score. ΔHAZ=change in HAZ (ie, HAZ at follow–up visit [50–90 days after enrolment] minus HAZ at enrolment). LSD=less severe diarrhoea. MSD=moderate–to–severe diarrhoea.

We examined patient management according to clinical syndrome. While at the sentinel hospital or health centre, oral rehydration salts were given to 471 (19·9%) of 2345 children with MSD and 74 (2·3%) of 3174 children with LSD; by contrast, most children (5133 [92·6%] of 5542) received a prescription for oral rehydration salts to be administered at home. A prescription for zinc was given to 2601 (46·9%) of 5542 children. At the sentinel-hospital or health-centre visit, antibiotics were administered to 93 (12·7%) of 730 children with dysentery, to 281 (17·2%) of 1638 children with non-dysentery MSD, and to 38 (1·2%) of 3174 children with LSD; 557 (76·3%) of 730 children with dysentery, 573 (34·9%) of 1638 children with non-dysentery MSD, and 1788 (56·3%) of 3174 children with LSD received a prescription for antibiotics for administration at home after discharge from the sentinel hospital or health centre.

## Discussion

Results from this study corroborate several important observations from GEMS about the aetiology and adverse clinical consequences of MSD among children under 5 years of age living in low-income communities in sub-Saharan Africa and south Asia, and extend those findings to a much broader population of children.^2^ GEMS demonstrated that four pathogens (rotavirus, *Cryptosporidium* spp, enterotoxigenic *E coli* producing heat-stable toxin, and *Shigella* spp*)* were responsible for the majority of attributable MSD cases. Inclusion of LSD in the current study revealed a far greater burden contributed by these pathogens. By estimating the attributable fraction and the attributable incidence of pathogen-specific MSD and LSD, our data not only show the proportion of episodes that might be prevented by using an effective intervention, but also characterise the public health impact that such an intervention might have.^17^ Even if a vaccine were less effective against LSD than MSD, as has been the case for rotavirus vaccines,^18^ the vaccine would be expected to prevent many more episodes of LSD than of MSD.

Children with MSD in general were not more stunted at baseline than those with LSD. Thus, we cannot attribute the increased severity of their diarrhoeal illness to their pre-existing linear growth faltering. Moreover, despite evident differences in illness severity, children with both syndromes demonstrated linear growth faltering relative to their matched healthy controls during the 2–3 months after their illness. These findings illustrate the importance of preventing both LSD and MSD from the perspective of mitigating the adverse nutritional consequences of young-child diarrhoeal illness.

This study also provided a broad view of the patient management of diarrhoeal disease at health centres in Africa and Asia. The purported trends toward diminishing prioritisation and funding of diarrhoea control programmes in low-income and middle-income countries^19^ are perhaps reflected in the suboptimal centre-based administration of oral rehydration salts at our sites. Few children actually received these fluids during their visit and instead oral rehydration salts were prescribed at the sentinel hospital or health centre but left to the child's caretakers to procure and administer at home. Without substantial investment of resources to support intense promotion of oral rehydration salt use at the community level, it is unlikely that the full public health impact of oral rehydration salts will be realised.^19^ Moreover, it is uncertain whether oral rehydration salts and zinc alone will be sufficient to prevent growth faltering and reduce diarrhoea-related fatalities that we have found to be associated with several important causes of MSD and LSD in low-resource settings, such as *Cryptosporidium* spp, enterotoxigenic *E coli* producing heat-stable toxin, and *Shigella* spp,^2^ which induce intestinal pathology in addition to fluid loss. The development and evaluation of strategies for prevention and treatment of these three pathogens for use in low-resource settings and the impact of these interventions on the acute and longer-term consequences of diarrhoea should be research priorities.

Our case-control study identified an association between *H pylori* and diarrhoeal disease (both MSD and LSD) at multiple sites, affecting all age groups, and at all three Asian sites and at the Mali site. Our findings are supported by a longitudinal study of Peruvian children aged 6 months to 12 years that observed an increased incidence of diarrhoea during the first 2 months after acquisition of acute *H pylori* infection.^20^ Although both studies raise the prospect that *H pylori* is a diarrhoeal pathogen, one cannot exclude the possibility that it is a modifier that increases susceptibility to certain other enteropathogens, or a co-traveller that shares risk factors with other enteric infections, such as contaminated drinking water, household crowding, and inadequate hygiene.^21^ Studies that support the modifier theory, perhaps mediated by the propensity of persistent *H pylori* infection to cause hypochlorhydria, have found that children and adults with *H pylori* infection had an increased risk of typhoid fever in India^22^ and an increased severity (but not risk) of cholera in Bangladesh.^23^ Evidence shedding doubt on the diarrhoeagenicity of *H pylori* includes findings that seronegative US adults who were challenged with *H pylori* did not report diarrhoeal symptoms.^24^, ^25^, ^26^ Additionally, three prospective studies among children younger than 5 years in developing countries did not find an overall increase in the incidence of diarrhoea in those with *H pylori* infection; however, methodological issues such as enrolment of children with pre-existing infection confound the interpretation of these findings.^27^, ^28^, ^29^

In many respects, the findings of GEMS-1A (and its parent study GEMS^2^) are complemented by the Malnutrition and Enteric Disease (MAL-ED) study,^30^ a contemporaneous multicentre, longitudinal, community-based cohort study of enteric infections among infants aged 0–23 months in eight low-income and middle-income countries. Both studies measured the proportion of diarrhoeal disease that was attributable to a broad array of pathogens, adjusting for asymptomatic detection of pathogens in controls, and both evaluated the impact of illness on growth and mortality. By design, children enrolled in GEMS and GEMS-1A were more severely ill, inhabited more impoverished environments, and spanned a broader age range, as detailed in a comparison published elsewhere.^31^ A minority (25·0%) of MAL-ED cases met the definition of LSD (ie, sought care at a health centre but were not considered MSD) and only 10·2% met criteria for MSD.^32^ Accordingly, compared with mortality among children in MAL-ED (0·05%)^32^ mortality in GEMS-1A was ten times higher among children with LSD and 20 times higher among children with MSD (unpublished). Moreover, the small reductions in HAZ (<-0·025) at 3 months associated with diarrhoea in MAL-ED^33^ were substantially lower than those seen among similarly aged children after LSD and MSD in GEMS-1A. These data illustrate the greater morbidity and mortality associated with the medically attended, more clinically severe diarrhoeal diseases included in GEMS-1A than with the milder episodes identified in the community during MAL-ED, and could help to explain the absence of association between diarrhoeal disease and growth faltering observed in MAL-ED.^33^ The relative contribution of various pathogens to the attributable disease burden could also contribute to the degree of growth faltering observed. Keeping in mind that cross-study comparisons must be interpreted with caution, it appears that a broader spectrum of pathogens (assessed with conventional assays because quantitative PCR was not used in GEMS-1A) is associated with the less severe diarrhoeal episodes in GEMS-1A and MAL-ED,^30^ whereas fewer, and perhaps more pathogenic organisms (eg, rotavirus, *Cyptosporidium* spp, and *Shigella* spp), were associated with a greater proportion of the more severe MSD episodes.

Several limitations must be considered when interpreting the results of this study. For one, enrolment was undertaken for only one calendar year, thus limiting statistical power for comparisons. The method for calculating incidence by using the proportion of children with MSD or LSD taken to one of the site's sentinel hospitals or health centres to derive overall and pathogen-specific population-based incidence values assumes that pathogen distribution is similar in participants who seek and do not seek care at a sentinel hospital or health centre. This is a limitation particularly when applied to LSD. In the absence of unique clinical parameters to define LSD, incident cases included all episodes of diarrhoea occurring in the community that were not MSD, which encompass a range of severity. If LSD episodes seen at the sentinel hospital or health centre were more severe than those of children not seeking care at the sentinel hospital or health centre, then the pathogen-specific incidence values that were calculated on the basis of the pathogens identified among LSD cases at the sentinel hospital or health centre might be over-represented. It is thus reassuring that the proportion of children with LSD in the community who met WHO criteria for dehydration was similar to that seen among patients with LSD who sought care at the sentinel hospital or health centre. Moreover, our estimates of the overall incidence of acute, new-onset diarrhoeal disease (ie, MSD plus LSD) were considerably lower than reported elsewhere, so our measurements might actually represent underestimates.^34^ Finally, parents participating in the health-care utilisation and attitudes surveys probably under-reported milder diarrhoeal episodes occurring during the previous 7 days.^7^, ^35^

In summary, our results show that the same four pathogens—ie, rotavirus, *Cryptosporidum* spp, *Shigella* spp, and enterotoxigenic *E coli* producing heat-stable toxin—are responsible for most episodes of MSD and LSD. Our findings markedly expand the numbers of children adversely affected nutritionally by the consequences of diarrhoeal diseases but do not alter the focus of preventive efforts. The development and evaluation of strategies for prevention and treatment of *Cryptosporidium* spp, *Shigella* spp, and enterotoxigenic *E coli* producing heat-stable toxin for use in low-resource settings and the impact of these interventions on the acute and long-term consequences of diarrhoea should be priorities for future investigations.
