# Supplementary material for: The incidence, aetiology, and adverse clinical consequences of less severe diarrhoeal episodes among infants and children residing in low-income and middle-income countries: a 12-month case-control study as a follow-on to the Global Enteric Multicenter Study (GEMS)
Source: Lancet Glob Health. 2019 Apr 15;7(5):e568–84. doi: 10.1016/S2214-109X(19)30076-2 (PMC6484777; doi:10.1016/S2214-109X(19)30076-2)

THE LANCET
Global Health

Supplementary appendix

This appendix formed part of the original submission and has been peer reviewed. We post it as supplied by the authors.

Supplement to: Kotloff KL, Nasrin D, Blackwelder WC, et al. The incidence, aetiology, and adverse clinical consequences of less severe diarrhoeal episodes among infants and children residing in low-income and middle-income countries: a 12-month case-control study as a follow-on to the Global Enteric Multicenter Study (GEMS). *Lancet Glob Health* 2019; **7:** e568–84.

PCR	Target	Primer	Primer sequence (5’ to 3’)	Amplicon (bp)
Duplex	eae	eae-384-F	GACCCGGCACAAGCATAAGC	384
		eae-384-R	CCACCTGCAGCAACAAGAGG	
	STp	STp-F	TCTTTCCCCTCTTTTAGTCAG	166
		STp-R	ACAGGCAGGATTACAACAAAG	
Monoplex	*bfpA*	BFPA-F	GGAAGTCAAATTCATGGGGG	300
		BFPA-R	GGAATCAGACGCAGACTGGT	
Multiplex #2	*efa-1*	Efa-1-F	CCATATGACAACGACAATGA	165
		Efa-1-R	TTCAGCTACAGGAGATCGTT	
	eae	eae-F-377	GGYCAGCGTTTTTTCCTTCCTG	377
		eae-R-377	TCGTCACCARAGGAATCGGAG	
	stx1	Stx-1-F	TGAATGTCATTCGCTCTGCAA	220
		Stx-1-R	TGTGAAAAATCAGCAAAGCGATAAAAA	
	stx2	Stx-2-F	GACCATCTTCGTCTGATTATTGAGC	443
		Stx-2-R	TACTCCGGAAGCACATTGC	
	sen	Sen-F	ACAAATCGGCATCAAATTAT	310
		Sen-R	TATATAACGCTTCCCCAAGA	

Supplemental Table 1. Duplex, monoplex and multiplex #2 PCRs used in this study.

	Basse, The Gambia		Bamako, Mali		Manhiça, Mozambique		Kolkata, India		Mirzapur, Bangladesh		Karachi (Bin Qasim Town), Pakistan	
	Cases	Controls	Cases	Controls	Cases	Controls	Cases	Controls	Cases	Controls	Cases	Controls
**0-11 months**												
N	220	260	236	236	155	155	213	213	183	366	227	228
Mean age in months (SD)	6·9 (2·7)	7·1 (2·5)	6·7 (2·8)	6·7 (2·6)	7·0 (2·5)	7·3* (2·5)	5·9 (2·9)	5·8 (2·7)	8·0 (2·2)	7·5* (2·2)	5·9 (2·9)	5·7 (2·9)
No. of females (%)	103 (47%)	120 (46%)	105 (44%)	105 (44%)	72 (46%)	72 (46%)	89 (42%)	89 (42%)	86 (47%)	172 (47%)	111 (49%)	111 (49%)
No. caretaker completed primary school (%)	31 (14%)	27 (10%)	39 (17%)	59* (25%)	53 (34%)	58 (37%)	151 (71%)	161 (76%)	150 (82%)	301 (82%)	57 (25%)	39 (17%)*
Mean wealth quintile (SD)	1·9 (1·4)	2·1 (1·3)	1·9 (1·5)	2·0 (1·4)	1·9 (1·5)	2·0 (1·3)	2·2 (1·4)	2·0 (1·4)	2·0 (1·4)	2·0 (1·5)	1·9 (1·4)	2·3* (1·4)
Median no. in household (range)	24·5 (3-87)	32·5* (3-138)	13 (3-74)	14 (3-67)	6·7 (2-22)	7·9* (2-66)	6 (3-13)	6 (3-21)	5·4 (2-15)	5·8 (2-16)	8 (3-26)	8 (2-26)
No. access to improved water (%)	104 (47%)	146 (56%)	158 (67%)	156 (66%)	55 (35%)	53 (34%)	41 (19%)	63 (30%)*	183 (100%)	366 (100%)	4 (2%)	0 (0%)
**12-23 months**												
N	202	273	226	227	175	175	180	194	148	296	171	309
Mean age in months (SD)	17·2 (3·6)	17·2 (3·2)	17·1 (3·6)	17·3 (3·4)	16·1 (3·2)	16·5 (3·1)	17·1 (3·5)	17·2 (3·2)	15·9 (3·3)	16·2 (3·0)	16·8 (3·5)	16·6* (2·9)
No. of females (%)	93 (46%)	129 (47%)	123 (54%)	124 (55%)	75 (43%)	75 (43%)	81 (45%)	87 (45%)	66 (45%)	132 (45%)	83 (49%)	149 (48%)
No. caretaker completed primary school (%)	25 (12%)	19 (7%)*	36 (16%)	42 (19%)	63 (36%)	53 (30%)	122 (68%)	142 (73%)	124 (84%)	241 (81%)	29 (17%)	59 (19%)
Mean wealth quintile (SD)	2·1 (1·4)	2·1 (1·4)	2·1 (1·4)	2·1 (1·4)	1·9 (1·4)	2·0 (1·4)	1·7 (1·4)	2·1* (1·5)	2·0 (1·4)	1·9 (1·4)	1·8 (1·4)	2·0 (1·4)
Median no. in household (range)	25 (2-100)	33* (3-130)	15 (3-99)	14 (3-77)	6 (2-22)	7* (2-18)	6 (2-22)	6 (3-19)	5·7 (2-17)	5·2* (2-14)	8 (3-40)	7 (3-25)
No. access to improved water (%)	99 (49%)	134 (49%)	151 (67%)	155 (68%)	69 (39%)	51 (29%)*	26 (14%)	56 (29%)*	147 (99%)	294 (99%)	2 (1%)	3 (1%)

**24-59 months**												
N	135	250	230	230	101	101	181	187	83	248	108	288
Mean age in months (SD)	32·0 (8·4)	31·8* (7·5)	35·3 (8·9)	35·3 (8·6)	33·4 (8·2)	34·0 (7·9)	38·6 (10·7)	38·4 (10·5)	35·0 (9·2)	35·2 (8·9)	36·1 (10·0)	35·9 (9·5)
No. of females (%)	75 (56%)	143 (57%)	115 (50)	115 (50)	48 (48%)	48 (48%)	92 (51%)	93 (50%)	25 (30%)	75 (30%)	49 (45%)	129 (45%)
No. caretaker completed primary school (%)	13 (10%)	14 (6%)	32 (14%)	45 (20%)	27 (27%)	34 (34%)	115 (64%)	112 (60%)	71 (86%)	186 (75%)*	23 (21%)	72 (25%)
Mean wealth quintile (SD)	2·0 (1·4)	2·0 (1·3)	1·9 (1·4)	2·1 (1·4)	2·0 (1·6)	2·1 (1·4)	1·9 (1·4)	1·8 (1·4)	2·1 (1·5)	1·9 (1·5)	1·7 (1·2)	2·3* (1·4)
Median no. in household (range)	25 (3-84)	35* (4-120)	14 (3-58)	15 (3-77)	6 (3-40)	6 (2-98)	6 (3-13)	5* (3-13)	5 (2-12)	5 (2-26)	7 (3-20)	8 (3-35)
No. access to improved water (%)	67 (50%)	140 (56%)	177 (77%)	148 (64%)*	35 (35%)	29 (29%)	26 (14%)	38 (20%)	83 (100%)	245 (99%)	0 (0%)	2 (1%)

SD=Standard deviation. *p<0·05 by Wald Chi-Square test

Supplemental Table 1. Demographic features of cases with less severe diarrhoea and their matched controls.

	Basse, The Gambia		Bamako, Mali		Manhiça, Mozambique		Kolkata, India		Mirzapur, Bangladesh		Karachi (Bin Qasim Town), Pakistan	
	Cases	Controls	Cases	Controls	Cases	Controls	Cases	Controls	Cases	Controls	Cases	Controls
**0-11 months**												
N	124	201	234	234	65	187	206	207	122	244	155	155
Mean age in months (SD)	7.8 (2.2)	7.8 (2.2)	7.4 (2.5)	7.3 (2.6)	7.1 (3.0)	7.3 (2.6)	6.5 (3.0)	6.2* (2.8)	7.8 (2.4)	7.5* (2.3)	7.0 (2.8)	6.8* (2.6)
No. of females (%)	44 (35%)	61 (30%)	125 (53.4%)	125 (53.4%)	25 (38.5%)	73 (39.0%)	89 (43.2%)	90 (43.5%)	47 (38.5%)	94 (38.5%)	75 (48.4%)	75 (48.4%)
No. caretaker completed primary school (%)	5 (4%)	11 (5%)	53 (22.7%)	45 (19.2%)	25 (38.5%)	61 (32.6%)	142 (68.9%)	145 (70.1%)	102 (83.6%)	202 (82.8%)	30 (19.4%)	29 (18.7%)
Mean wealth quintile (SD)	1.6 (1.4)	2.0* (1.4)	2.0 (1.4)	2.1 (1.4)	2.3 (1.4)	2.0 (1.4)	2.0 (1.4)	2.1 (1.4)	2.0 (1.3)	2.0 (1.4)	1.9 (1.4)	2.0 (1.5)
Median no. in household (range)	23.5 (4-90)	34.0* (3-116)	14.0 (3-79)	14.0 (2-62)	7.0 (2-25)	7.0 (2-29)	6.0 (3-31)	6.0 (3-17)	5.0 (3-30)	5.0 (3-15)	8.0 (3-29)	7.0 (2-30)
No. access to improved water (%)	60 (48%)	99 (49%)	156 (66.7%)	156 (66.7%)	26 (40.0%)	55 (29.4%)	40 (19.4%)	60* (29.0%)	121 (99.2)	242 (99.2%)	2 (1.3%)	1 (0.7%)
**12-23 months**												
N	159	258	229	229	43	127	164	180	103	206	113	226
Mean age in months (SD)	17.2 (3.3)	17.6 (3.2)	16.8 (3.6)	17.1 (3.3)	16.3 (3.4)	16.7 (3.2)	16.4 (3.5)	16.7 (3.1)	17.3 (3.4)	17.1 (3.1)	16.4 (3.4)	16.1 (2.9)
No. of females (%)	74 (47%)	111 (43%)	86 (37.6%)	86 (37.6%)	18 (41.9%)	54 (42.5%)	74 (45.1%)	80 (44.4%)	36 (35.0%)	72 (35.0%)	47 (41.6%)	94 (41.6%)
No. caretaker completed primary school (%)	9 (6%)	14 (5%)	46 (20.1%)	51 (22.3%)	14 (32.6%)	49 (38.6%)	105 (64.0%)	125 (69.4%)	85 (82.5%)	156 (75.7%)	20 (17.7%)	51 (22.6%)
Mean wealth quintile (SD)	2.1 (1.5)	2.0 (1.4)	2.0 (1.4)	2.2 (1.4)	2.3 (1.3)	2.0 (1.4)	1.8 (1.5)	2.1 (1.5)	2.0 (1.4)	2.0 (1.5)	1.6 (1.4)	1.9 (1.5)
Median no. in household (range)	29.0 (3-192)	33.0 (6-154)	12 (3-89)	16 (3-51)	6.0 (3-13)	7.0 (2-16)	5.0 (2-18)	5.0 (3-16)	5.0 (2-14)	5.0 (2-18)	8.0 (3-29)	7.0 (3-60)
No. access to improved water (%)	72 (45%)	131 (51%)	163 (71.2%)	158 (69.0%)	17 (39.5%)	35 (27.6%)	32 (19.5%)	50* (27.8%)	103 (100%)	206 (100%)	1 (0.9%)	6 (2.7%)
**24-59 months**												

N	69	158	221	221	25	71	169	192	95	285	72	216
Mean age in months (SD)	30.7 (7.5)	31.6* (7.2)	35.7 (8.8)	35.9 (9.1)	32.7 (8.8)	33.2 (8.6)	36.7 (9.4)	36.4 (9.4)	35.5 (9.4)	35.8 (9.2)	34.8 (9.0)	34.8 (8.6)
No. of females (%)	42 (61%)	101 (64%)	100 (45.3%)	100 (45.3%)	7 (28.0%)	19 (26.8%)	94 (55.6%)	111 (57.8%)	38 (40.0%)	114 (40.0%)	35 (48.6%)	105 (48.6%)
No. caretaker completed primary school (%)	10 (14%)	13 (8%)	44 (19.9%)	40 (18.1%)	7 (28.0%)	19 (26.8%)	107 (63.3%)	139* (72.4%)	71 (74.7%)	224 (78.6%)	10 (13.9%)	52 (24.1%)
Mean wealth quintile (SD)	2.2 (1.5)	1.9 (1.4)	1.7 (1.4)	2.0* (1.4)	1.8 (1.6)	1.8 (1.4)	1.7 (1.4)	2.1* (1.5)	1.9 (1.4)	2.0 (1.4)	1.6 (1.4)	2.1* (1.4)
Median no. in household (range)	33.0 (3-150)	32 (5-113)	13 (3-79)	14 (3-71)	5 (2-14)	6 (3-16)	6.0 (3-30)	5.0 (3-14)	5.0 (2-16)	5.0 (2-17)	8.0 (3-30)	8.0 (3-24)
No. access to improved water (%)	31 (45%)	78 (49%)	157 (71.0%)	150 (67.9%)	7 (28.0%)	20 (28.2%)	25 (14.8%)	40* (20.8%)	95 (100%)	285 (100%)	1 (1.4%)	1 (0.5%)

SD=Standard deviation. *p<0.05 by Wald Chi-Square test

Supplemental Table 2. Demographic features of cases with moderate-to-severe diarrhoea and their matched controls.

	GEMS 1a MSD			HUAS GEMS 1a MSD						GEMS 1a LSD			HUAS GEMS 1a LSD					
				Sought care at SHC			DID NOT seek care at SHC						Sought care at SHC			DID NOT seek care at SHC		
**0-11 months**	**No.+/ Total**	**%**	**95% CI**	**No.+/ Total**	**%**	**95% CI**	**No.+/ Total**	**%**	**95% CI**	**No.+/ Total**	**%**	**95% CI**	**No.+/ Total**	**%**	**95% CI**	**No.+/ Total**	**%**	**95% CI**
The Gambia	71/124	57.3	48.1 - 66.1	24/29	82.8	64.2 - 94.2	66/74	89.2	79.8 - 95.2	1/220	0.5	0 - 2.5	27/91	29.7	20.5 - 40.2	47/216	21.8	16.4 - 27.9
Mali	215/234	91.9	87.6 - 95	10/12	83.3	51.6 - 97.9	69/83	83.1	73.3 - 90.5	4/236	1.7	0.5 - 4.3	1/5	20.0	0.5 - 71.6	9/73	12.3	5.8 - 22.1
Mozambique	36/65	55.4	42.5 - 67.7	2/5	40.0	5.3 - 85.3	1 of 2	50.0	1.3 - 98.7	1/155	0.7	0 - 3.5	0/9	0	0 - 33.6	0/20	0	0 - 16.8
India	205/206	99.5	97.3 - 100	16/16	100	79.4 - 100	47/47	100	92.5 - 100	175/213	82.2	76.3 - 87.1	18/22	81.8	59.7 - 94.8	62/73	84.9	74.6 - 92.2
Bangladesh	5/122	4.1	1.3 - 9.3	1/4	25.0	0.6 - 80.6	2/5	40.0	5.3 - 85.3	0/183	0	0 - 2	0/2	0	0 - 84.2	0/18	0	0 - 18.5
Pakistan	119/155	76.8	69.3 - 83.2	7/8	87.5	47.3 - 99.7	44/48	91.7	80 - 97.7	32/227	14.1	9.8 - 19.3	9/33	27.3	13.3 - 45.5	27/148	18.2	12.4 - 25.4
**Total**	651/906	71.9	68.8 - 74.8	60/74	81.1	70.3 - 89.3	229/259	88.4	83.9 - 92	213/1234	17.3	15.2 - 19.5	55/162	34.0	26.7 - 41.8	145/548	26.5	22.8 - 30.4
**12-23 months**	**No.+/ Total**	**%**	**95% CI**	**No.+/ Total**	**%**	**95% CI**	**No.+/ Total**	**%**	**95% CI**	**No.+/ Total**	**%**	**95% CI**	**No.+/ Total**	**%**	**95% CI**	**No.+/ Total**	**%**	**95% CI**
The Gambia	72/159	45.3	37.4 - 53.4	29/34	85.3	68.9 - 95	87/94	92.6	85.3 - 97	1/202	0.5	0 - 2.7	28/92	30.4	21.3 - 40.9	42/199	21.1	15.7 - 27.4
Mali	203/229	88.7	83.8 - 92.4	9/10	90.0	55.5 - 99.7	60/78	76.9	66 - 85.7	4/226	1.8	0.5 - 4.5	1/3	33.3	0.8 - 90.6	3/35	8.6	1.8 - 23.1
Mozambique	17/43	39.5	25 - 55.6	0/4	0	0 - 60.2	1/3	33.3	0.8 - 90.6	1/175	0.6	0 - 3.1	0/9	0	0 - 33.6	0/16	0	0 - 20.6
India	164/164	100	97.8 - 100	8/8	100	63.1 - 100	37/38	97.4	86.2 - 99.9	162/180	90	84.7 - 94	12/14	85.7	57.2 - 98.2	59/71	83.1	72.3 - 91
Bangladesh	10/103	9.7	4.8 - 17.1	0/2	0	0 - 84.2	0/6	0	0 - 45.9	0/148	0	0 - 2.5	0/5	0	0 - 52.2	0/12	0	0 - 26.5
Pakistan	70/113	62	52.3 - 70.9	9/10	90.0	55.5 - 99.7	26/29	89.7	72.6 - 97.8	35/171	20.5	14.7 - 27.3	11/22	50.0	28.2 - 71.8	16/131	12.2	7.1 - 19.1
**Total**	536/811	66.1	62.7 - 69.3	55/68	80.9	69.5 - 89.4	211/248	85.1	80 - 89.3	203/1102	18.4	16.2 - 20.8	52/145	35.9	28.1 - 44.2	120/464	25.9	21.9 - 30.1
**24-59 months**	**No.+/ Total**	**%**	**95% CI**	**No.+/ Total**	**%**	**95% CI**	**No.+/ Total**	**%**	**95% CI**	**No.+/ Total**	**%**	**95% CI**	**No.+/ Total**	**%**	**95% CI**	**No.+/ Total**	**%**	**95% CI**
The Gambia	29/69	42	30.2 - 54.5	8/12	66.7	34.9 - 90.1	38/43	88.4	74.9 - 96.1	0/135	0	0 - 2.7	12 /42	28.6	15.7 - 44.6	23/138	16.7	10.9 - 24
Mali	193/221	87.3	82.2 - 91.4	4/7	57.1	18.4 - 90.1	26/35	74.3	56.7 - 87.5	0/230	0	0 - 1.6	0	0		6/26	23.1	9 - 43.6
Mozambique	4/25	16.0	4.5 - 36.1	1/2	50.0	1.3 - 98.7	1/2	50.0	1.3 - 98.7	1/101	1	0 - 5.4	0/4	0	0 - 60.2	0/7	0	0 - 41
India	167/169	98.8	95.8 - 99.9	2/2	100	15.8 - 100	24/24	100	85.8 - 100	153/181	84.5	78.4 - 89.5	14/14	100	76.8 - 100	51/57	89.5	78.5 - 96
Bangladesh	1/95	1.1	0 - 5.7	0	0		0/1	0	0 - 97.5	0/83	0	0 - 4.3	0/1	0	0 - 97.5	0/9	0	0 - 33.6

Pakistan	38/72	52.8	40.7 - 64.7	8/10	80.0	44.4 - 97.5	15/16	93.8	69.8 - 99.8	10/108	9.3	4.5 - 16.4	5/15	33.3	11.8 - 61.6	14/67	20.9	11.9 - 32.6
**Total**	432/651	66.4	62.6 - 70	23/33	69.7	51.3 - 84.4	104/121	86.0	78.5 - 91.6	164/838	19.6	16.9 - 22.4	31/76	40.8	29.6 - 52.7	94/304	30.9	25.8 - 36.4

Supplemental Table 3. Number (%) of cases who met the World Health Organization criteria for some/severe dehydration according to care seeking behavior in the GEMS 1a case control study and the GEMS 1a Health Care Utilization and Attitudes Survey (HUAS).

Supplemental Figure 1. Enrollment of cases with moderate-to-severe diarrhea (MSD) and less-severe diarrhea (LSD) by age.

(A) 0-11 months

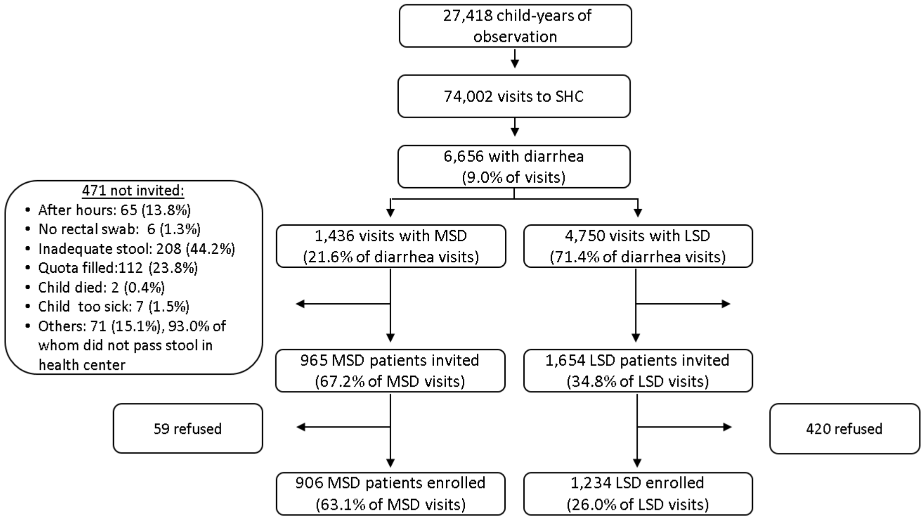

(B) 12-23 months

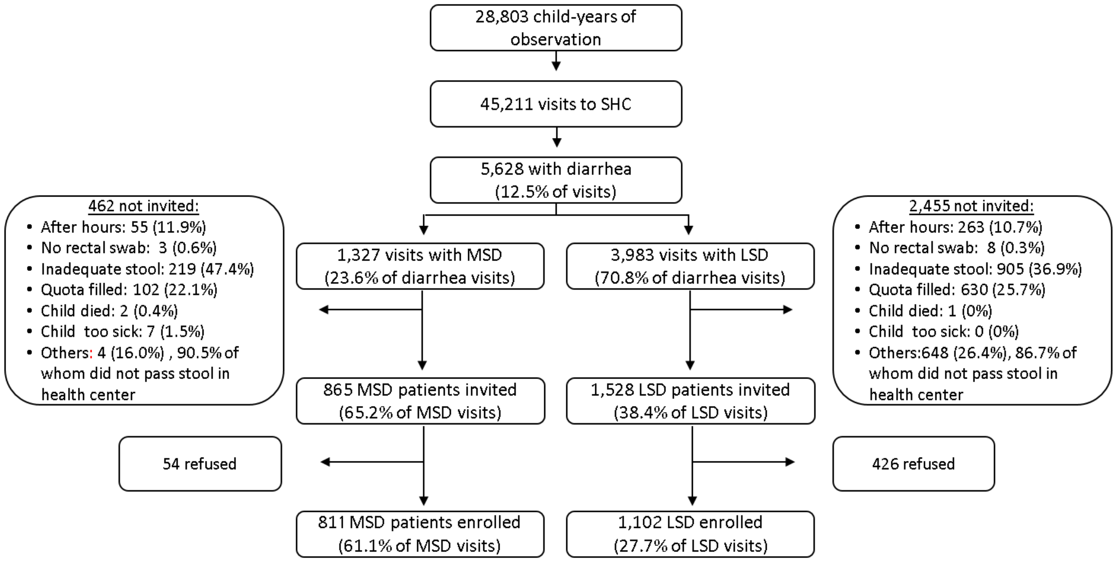

(C) 24-59 months

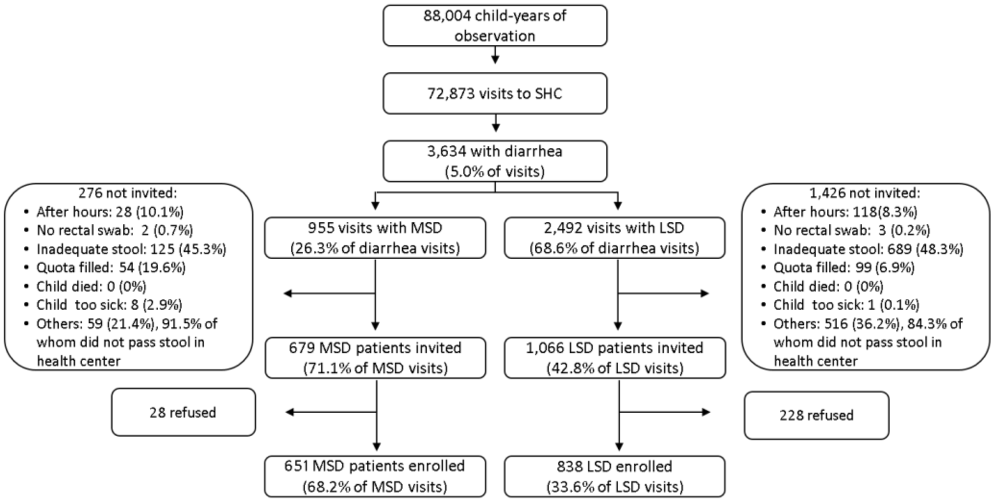

Supplement: Supplementary appendix [file mmc1.pdf]
